# Transmission and pathogenicity of canine H3N2 influenza virus in dog and guinea pig models

**DOI:** 10.1186/s12985-022-01888-x

**Published:** 2022-10-12

**Authors:** Ratanaporn Tangwangvivat, Supassama Chaiyawong, Nutthawan Nonthabenjawan, Kamonpan Charoenkul, Taveesak Janethanakit, Kitikhun Udom, Sawang Kesdangsakonwut, Rachod Tantilertcharoen, Aunyaratana Thontiravong, Alongkorn Amonsin

**Affiliations:** 1 Center of Excellences for Emerging and Re-emerging Infectious Diseases in Animals and One Health Research Cluster, Faculty of Veterinary Science, Chulalongkorn University, 10330 Bangkok, Thailand; 2 Department of Veterinary Public Health, Faculty of Veterinary Science, Chulalongkorn University, Bangkok, Thailand; 3 Division of Communicable Diseases, Department of Disease Control, Ministry of Public Health, Nonthaburi, Thailand; 4 Department of Pathology, Faculty of Veterinary Science, Chulalongkorn University, Bangkok, Thailand; 5 Department of Microbiology, Faculty of Veterinary Science, Chulalongkorn University, Bangkok, Thailand

**Keywords:** Canine influenza, Dog, Guinea pigs, H3N2, Pathogenicity, Transmission

## Abstract

**Background:**

Influenza A virus causes respiratory disease in many animal species as well as in humans. Due to the high human-animal interface, the monitoring of canine influenza in dogs and the study of the transmission and pathogenicity of canine influenza in animals are important.

**Methods:**

Eight-week-old beagle dogs *(Canis lupus familaris)* (n = 13) were used for the intraspecies transmission model. The dogs were inoculated intranasally with 1 ml of 10^6^ EID_50_ per ml of canine H3N2 influenza virus (A/canine/Thailand/CU-DC5299/2012) (CIV-H3N2). In addition, 4-week-old guinea pigs (*Cavia porcellus)* (n = 20) were used for the interspecies transmission model. The guinea pigs were inoculated intranasally with 300 µl of 10^6^ EID_50_ per ml of CIV-H3N2.

**Results:**

For the Thai CIV-H3N2 challenged in the dog model, the incoculated and direct contact dogs developed respiratory signs at 2 dpi. The dogs shed the virus in the respiratory tract at 1 dpi and developed an H3-specific antibody against the virus at 10 dpi. Lung congestion and histopathological changes in the lung were observed. For the Thai CIV-H3N2 challenge in the guinea pig model, the incoculated, direct contact and aerosol-exposed guinea pigs developed fever at 1–2 dpi. The guinea pigs shed virus in the respiratory tract at 2 dpi and developed an H3-specific antibody against the virus at 7 dpi. Mild histopathological changes in the lung were observed.

**Conclusion:**

The result of this study demonstrated evidence of intraspecies and interspecies transmission of CIV-H3N2 in a mammalian model.

## Background

Influenza virus causes respiratory disease in several animal species as well as in humans. The canine H3N8 influenza virus (CIV-H3N8), which originated from an equine H3N8 influenza virus, was first reported in racing grayhounds in the US in 2004. CIV-H3N8-infected dogs showed clinical signs of upper respiratory tract infection, such as cough, nasal discharge, fever and subsequent self-recovery [1, 2]. After the first outbreak, the CIV-H3N8 spread to several states in the US and the UK [3–5]. In 2008, avian-origin canine H3N2 influenza virus (CIV-H3N2) emerged in dogs in South Korea. CIV-H3N2 outbreaks were subsequently reported in dogs in southern China and Thailand [6, 7]. In 2015, CIV-H3N2 was also reported in the US [8].

Dog-to-dog transmission of CIV-H3N8 and CIV-H3N2 has been documented in dog shelters and animal hospitals [9–11]. In an animal experiment setting, CIV-H3N2-infected dogs develop influenza-like symptoms and shed virus [12]. The transmission of CIV-H3N2 from dogs to cats was reported in South Korea. Cats could be infected with CIV-H3N2 and developed respiratory signs in an experimental setting [13].

In influenza research, some mammalian species can be used as experimental models, such as ferrets, mice and guinea pigs. Ferrets are an excellent model for influenza and can transmit influenza virus naturally from infected to noninfected ferrets [14, 15]. However, the ferret model presents several disadvantages, such as its high cost and difficult restraint. As an alternative, the guinea pig can be used as a mammalian model for the study of influenza [16, 17]. The guinea pig is suitable for both large droplet and air-born influenza transmission in mammalian hosts [18]. The advantages are that they are inexpensive, easy to handle and susceptible to human influenza virus infection [19, 20]. In this study, the transmission and pathogenicity of Thai CIV-H3N2 (A/canine/Thailand/CU-DC5299/2012 (H3N2)) in dog and guinea pig models were investigated. Our results provide evidence of intraspecies and interspecies transmission of Thai CIV-H3N2 in a mammalian model.

## Methods

### Animals

This study was conducted under the ethical approval of the Chulalongkorn University Animal Care and Use Committee (CU-VET, IACUC protocol no. 13310032 and 1431100). Thirteen 8-week-old beagle dogs (*Canis lupus familaris*, n = 13) were used for transmission and pathogenicity studies. The animals were housed at the Biosafety Level 2 + facility for 14 days before the experiment. Dogs were tested and free from influenza antibody by the hemagglutinin inhibition test (HI) test with CIV-H3N2 before use. Twenty 4-week-old male Hartley strain guinea pigs (*Cavia porcellus*, n = 20) weighing 300 to 350 g were used for transmission and pathogenicity studies. The animals were obtained from the National Laboratory Animal Center, Bangkok, Thailand. Guinea pigs were housed at Biosafety Level 2 + for 14 days before the experiment. Guinea pigs were tested and confirmed to be influenza-free by the HI test before the experiment.

### Viruses

The canine H3N2 influenza virus (CIV-H3N2) used in this study was isolated from a dog with respiratory signs in Thailand in 2012. The virus, A/canine/Thailand/CU-DC5299/12 (H3N2), was previously characterized by whole-genome sequencing and was submitted to the Genbank (KC599545-52). In this study, the virus was propagated in embryonic chicken eggs to a concentration of 10^6^ EID_50_ per ml at the 4th passage level.

### Transmission and pathogenicity studies in a dog model

**Fig. 1:**
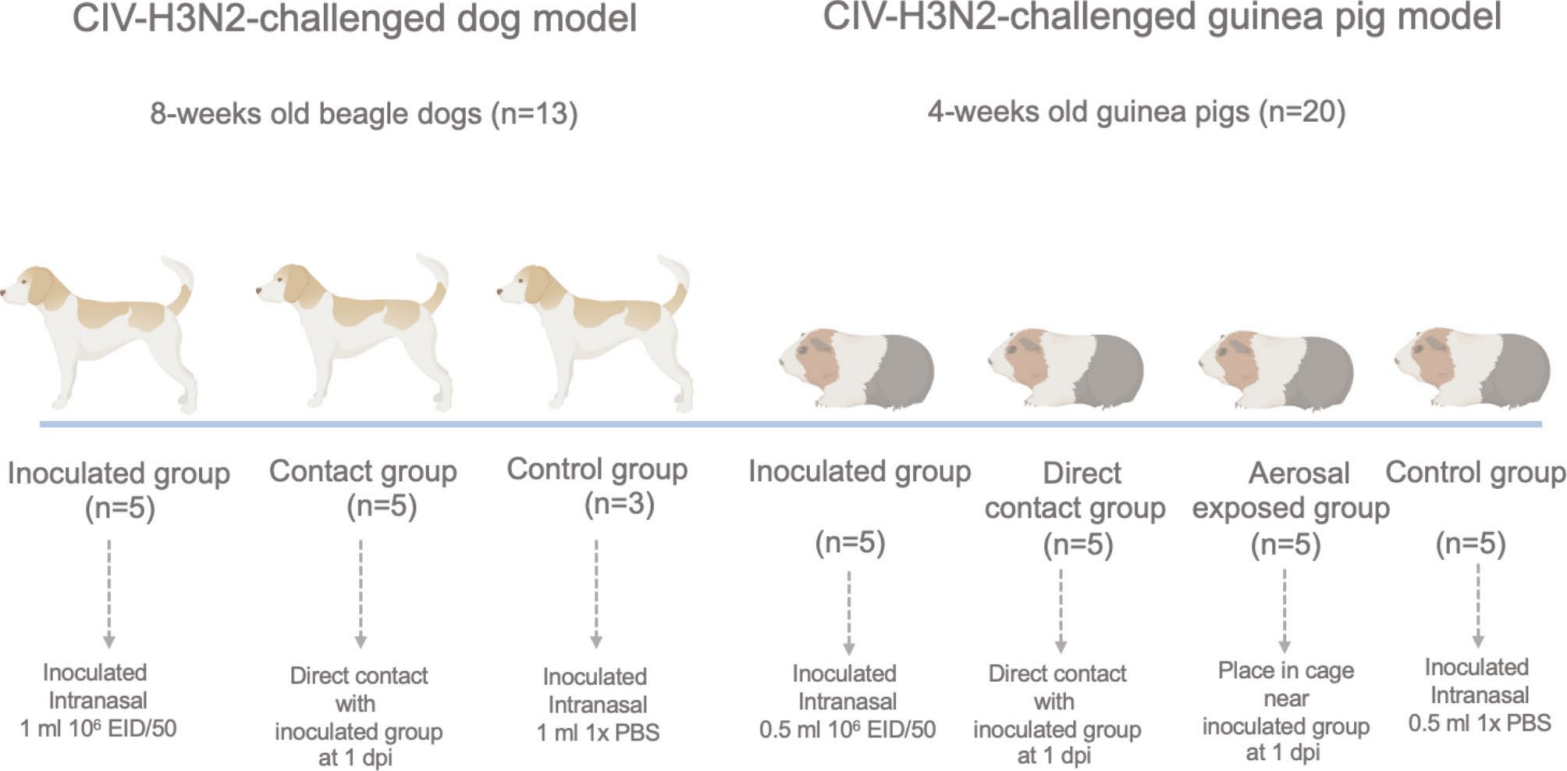
Study design of the transmission and pathogenicity of CIV-H3N2 in dog and guinea pig models

Dogs (n = 13) were randomly divided into 3 groups: the inoculated group (n = 5), the contact group (n = 5), and the control group (n = 3). Dogs were sedated with a mixture of xylazine and atropine (intramuscular administration). The inoculated group was inoculated intranasally with 1 ml (500 µl per nostril) of 10^6^ EID_50_ per ml of CIV-H3N2, whereas the control dogs were inoculated intranasally with 1 ml (500 µl per nostril) of phosphate buffer solution (PBS). In the contact group, dogs were placed in the same cage as inoculated dogs at 1 day post-inoculation (dpi). Measurements of CIV infection, transmission and pathogenicity in dogs, including clinical signs, viral shedding, antibody response and pathological changes, were recorded and analyzed. In detail, all dogs were observed daily for clinical signs, including body temperature, ocular discharge, nasal discharge, coughing, sneezing, panting, and abdominal breathing. Nasal swab samples of dogs in each group were collected daily at 1–10 dpi, 14 dpi and 21 dpi to quantitate viral shedding. Serum samples were collected at 7, 10, 14 and 21 dpi to monitor the antibody response. One dog from each group was randomly selected and euthanized at 7 and 14 dpi to observe pathological changes (gross and histopathological lesions) by hematoxylin and eosin (H&E) staining (Fig. 1).

### Transmission and pathogenicity studies in the guinea pig model

Twenty 4-week-old male Hartley strain guinea pigs (n = 20) were randomly divided into 4 groups, including the inoculated group (n = 5), direct contact group (n = 5), aerosol-exposed group (n = 5), and control group (n = 5). The inoculated group was inoculated intranasally with 300 µl of 10^6^ EID_50_ per ml of CIV-H3N2. The control group (n = 5) was inoculated intranasally with 300 µl of PBS. All guinea pigs were sedated with xylazine and ketamine (intramuscular administration). The guinea pigs of the direct contact group and aerosol-exposed group were placed 1 day after inoculation (1 dpi). For the aerosol-exposed group, the guinea pigs were placed 20 centimeters from the inoculated and direct contact groups. All guinea pigs were observed daily for clinical signs, including body temperature, ocular discharge, nasal discharge, coughing, sneezing, panting, and abdominal breathing. Nasal wash samples were collected at 1–7 dpi, 10 dpi and 14 dpi to quantitate viral shedding. Serum samples were collected at 7, 10 and 14 dpi to monitor the antibody response. One guinea pig from each group was randomly selected and euthanized at 3 dpi and 5 dpi to observe pathological changes (gross and histopathological lesions) using hematoxylin and eosin (H&E) staining (Fig. 1).

### Quantitation of viral concentration by real-time RT-PCR

Nasal swabs of dogs and nasal wash samples of guinea pigs were subjected to viral RNA extraction by a QIAamp Viral RNA Mini kit (Qiagen®; Hilden, Germany). Real-time RT-PCR was used for quantitation of viral concentration using the Superscript™ III Platinum® One-step Quantitative RT-PCR System (Invitrogen ™; California, USA) with an M gene-specific TagMan probe [21]. Rreal-time RT-PCR was performed on a Rotor-Gene 3000 (Corbett Research; Sydney, Australia) under the following conditions: 50 °C for 30 min, 95 °C for 15 min and 50 cycles of denaturing at 95 °C for 15 min and annealing/extension at 60 °C for 30 s. The data were analyzed using Rotor-Gene software, v.6.0.19. Ct values lower than 36 were considered positive. A standard curve of the copy number of viral RNA was constructed by ten-fold serial dilution and used for viral quantitation [22].

### Quantitation of H3-specific antibody by hemagglutinin inhibition (HI) assay

Blood samples of dogs and guinea pigs were centrifuged at 3,000 x g for 10 min to separate the serum. Serum samples were tested for H3-specific antibodies using a hemagglutinin inhibition (HI) assay. The HI protocol for canine serum samples was conducted as per the WHO recommendations (WHO, 2002). Briefly, receptor-destroying enzyme (RDE) was used to treat canine serum samples. Then, samples were absorbed with 50% chicken red blood cells and diluted into two-fold serial dilutions. Then, all treated samples were incubated at 25 °C for 45 min. Samples were added to a 0.5% suspension of chicken red blood cells. Then, 4 hemagglutination units per 25 µl of Thai CIV-H3N2 were added and incubated at room temperature for 1 h before HI titer interpretation. For the HI assay in guinea pigs, the assay was optimized following a previous study [23]. The guinea pig serum samples were treated with receptor destroying enzyme (RDE), absorbed with 1% turkey RBCs and then incubated with 4 HAU/25 µl of virus for 1 h before interpreting the results. An HI titer ≥ 40 was considered positive for both dog and guinea pig sera as previously described [23, 24].

### Statistical analysis

The descriptive statistics frequency and percentage were used to describe the clinical characteristics of dogs and guinea pigs. Analysis of significant differences in viral shedding and HI titers among groups was performed by independent t-test using Software for Statistics and Data Science (Stata) version 13.0. Graphs were plotted with Prism 8.

## Results

### Transmission and pathogenicity of Thai CIV-H3N2 in dogs

**Fig. 2:**
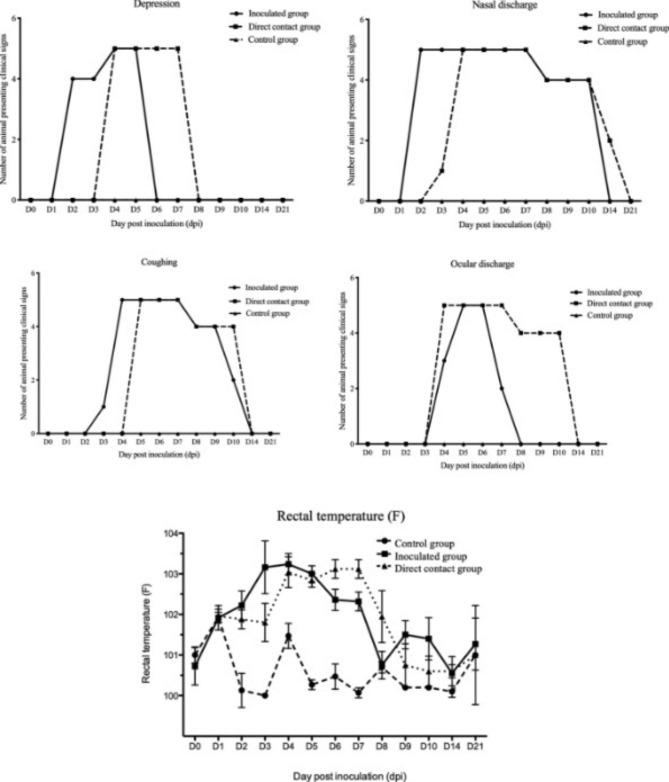
Clinical presentations and body temperature of CIV-H3N2-challenged dogs in the inoculated group, direct contact group and control group

Dogs in the inoculated group (n = 5) were challenged with 10^6^ EID_50_ of Thai CIV-H3N2, and the contact groups (n = 5) were placed after 1 dpi. PBS was used as a mock challenge in the control group (n = 3). For clinical presentation, all dogs in the inoculated group and direct contact group showed clinical signs, including fever, depression, nasal discharge, ocular discharge and coughing. In the control group, none of the dogs showed any clinical signs throughout the experiment. In detail, all dogs in the inoculated group and direct contact groups developed fever at 3 dpi and 4 dpi, respectively. Dogs in the inoculated group showed clinical signs from 2 dpi with mild depression, loss of appetite and less activity (2 dpi − 5 dpi) and serous nasal discharge (2 dpi − 10 dpi). One dog developed coughing at 3 dpi and the others presented coughing at 4 dpi − 10 dpi. Ocular discharge was observed at 4 dpi − 7 dpi. In the contact group, one dog (n = 1) showed serous nasal discharge at 3 dpi (3 dpi equal to 2 days post-contact), but the other dogs (n = 4) demonstrated clinical signs from 4 dpi to 14 dpi. Depression was also observed from 4 dpi to 7 dpi. All dogs in the contact group showed coughing at 5 dpi − 10 dpi. Ocular discharge was observed from 4 dpi to 10 dpi (Fig. 2).

**Fig. 3:**
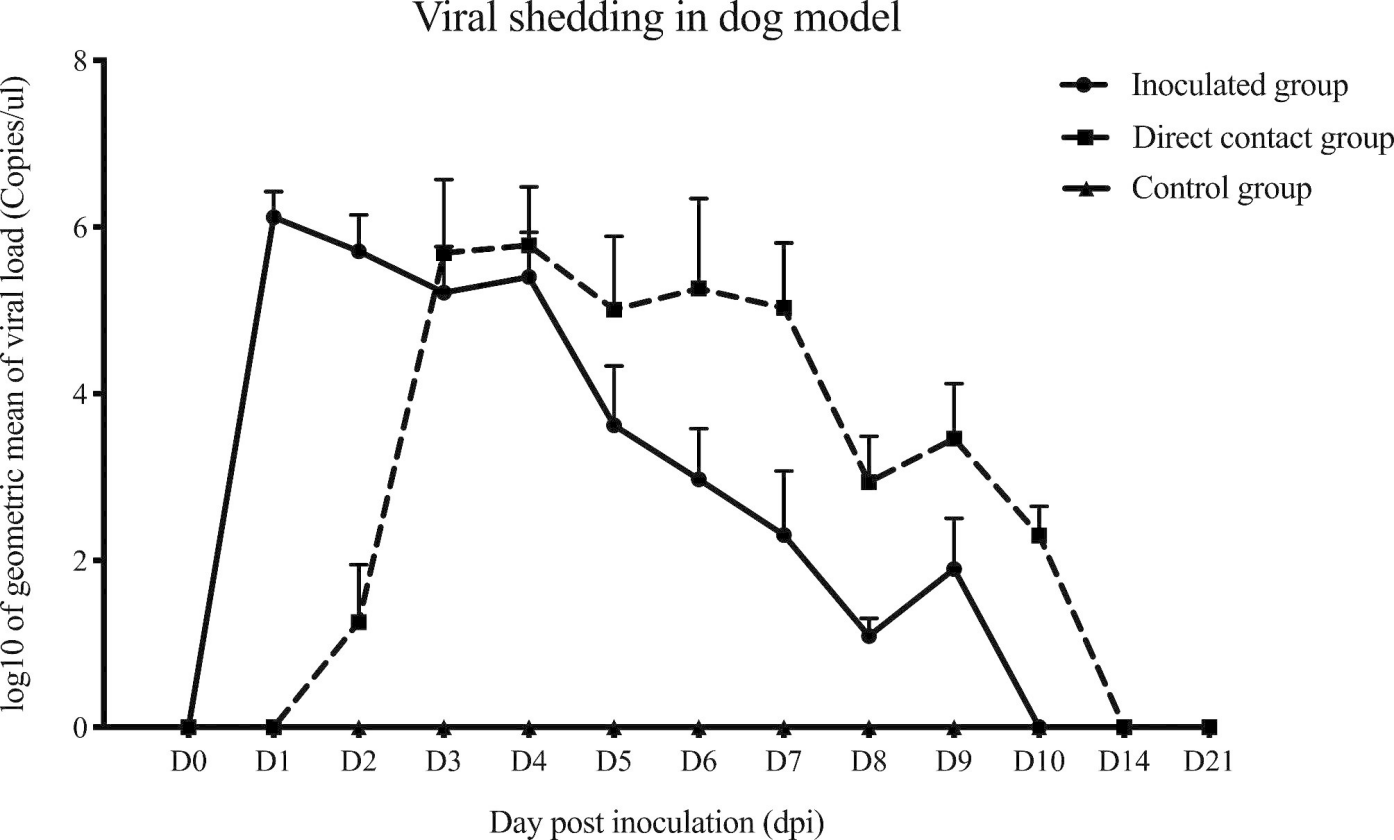
Viral shedding of CIV-H3N2-challenged dogs in the inoculated, direct contact and control groups. Viral shedding was presented as log10 of the geometric mean (copies per microliter). Bars represent the standard deviation of the mean viral titer

For viral shedding in dogs, nasal swab samples were collected on days − 1, 0, 10, 14 and 21 dpi. All dogs were negative for CIV-H3N2 before inoculation (-1 and 0 dpi). After challenge, CIV-H3N2 could be detected in the respiratory tract of dogs in both the inoculated group and the contact group. Dogs in the inoculated group shed the highest CIV-H3N2 in the respiratory tract at 1 dpi and decreased gradually until 9 dpi. In dogs in the contact group, some dogs shed the virus from 2 dpi (1 day post-contact) and the viral shedding was highest at 3 dpi and decreased gradually until 10 dpi. The viral shedding among the inoculated and contact groups was statistically significant (P < 0.05) (Fig. 3).

**Fig. 4:**
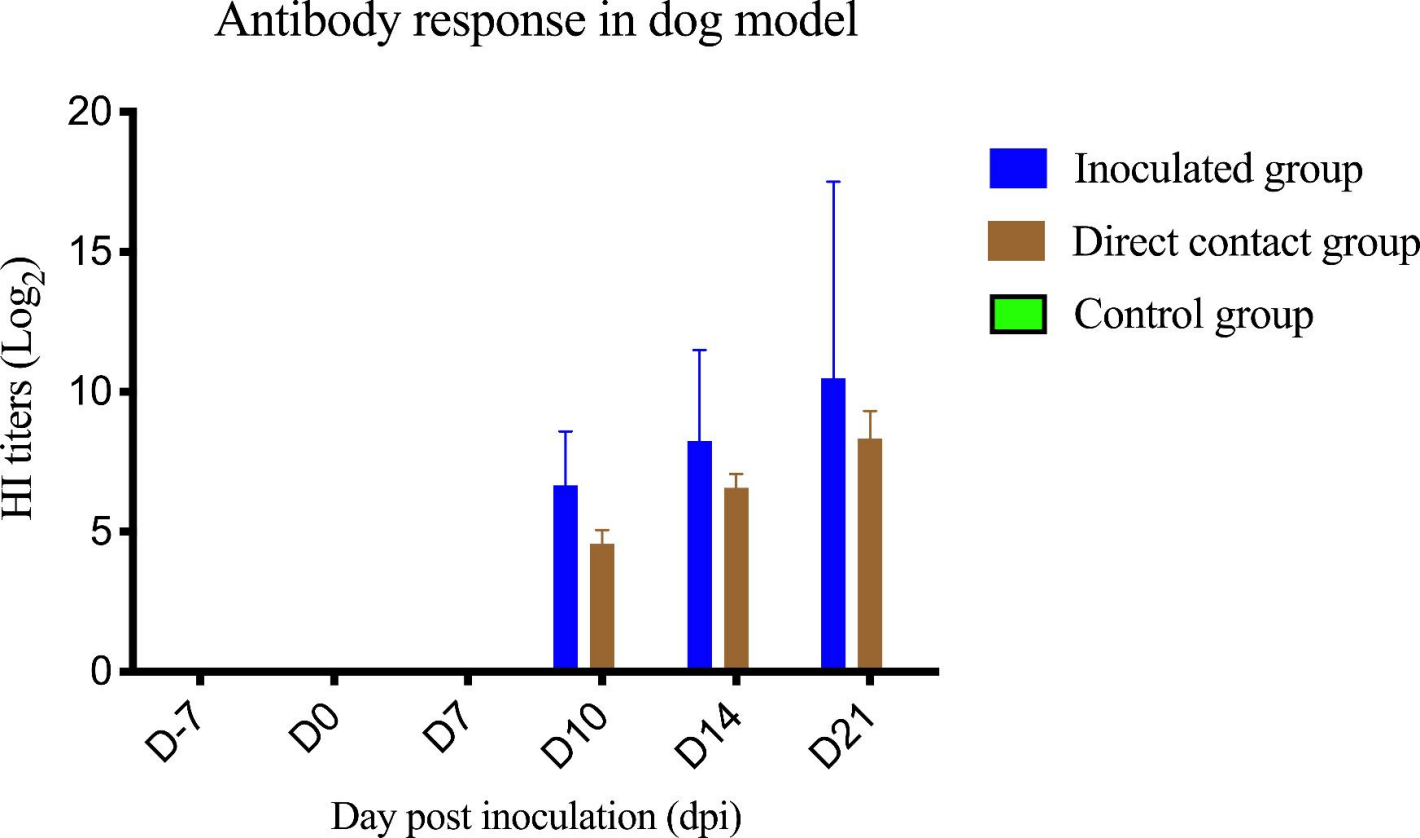
Antibody response (HI test) of CIV-H3N2-challenged dogs in the inoculated, direct contact and control groups. The antibody titer was presented as the HI titer. Bars represent the standard deviation of the mean HI titer

For the antibody response to CIV-H3N2, dogs in the inoculated and contact groups were seropositive at 10–21 dpi and 14–21 dpi, respectively. Our results suggested that HI antibody titers against Thai CIV-H3N2 developed at 10 dpi in the inoculated group and 14 dpi in the contact group. As expected, the dogs in the control group did not have HI antibody throughout the experiment. There were statistically significant differences (p < 0.05) in HI antibody titers among the groups (Fig. 4).

**Fig. 5:**
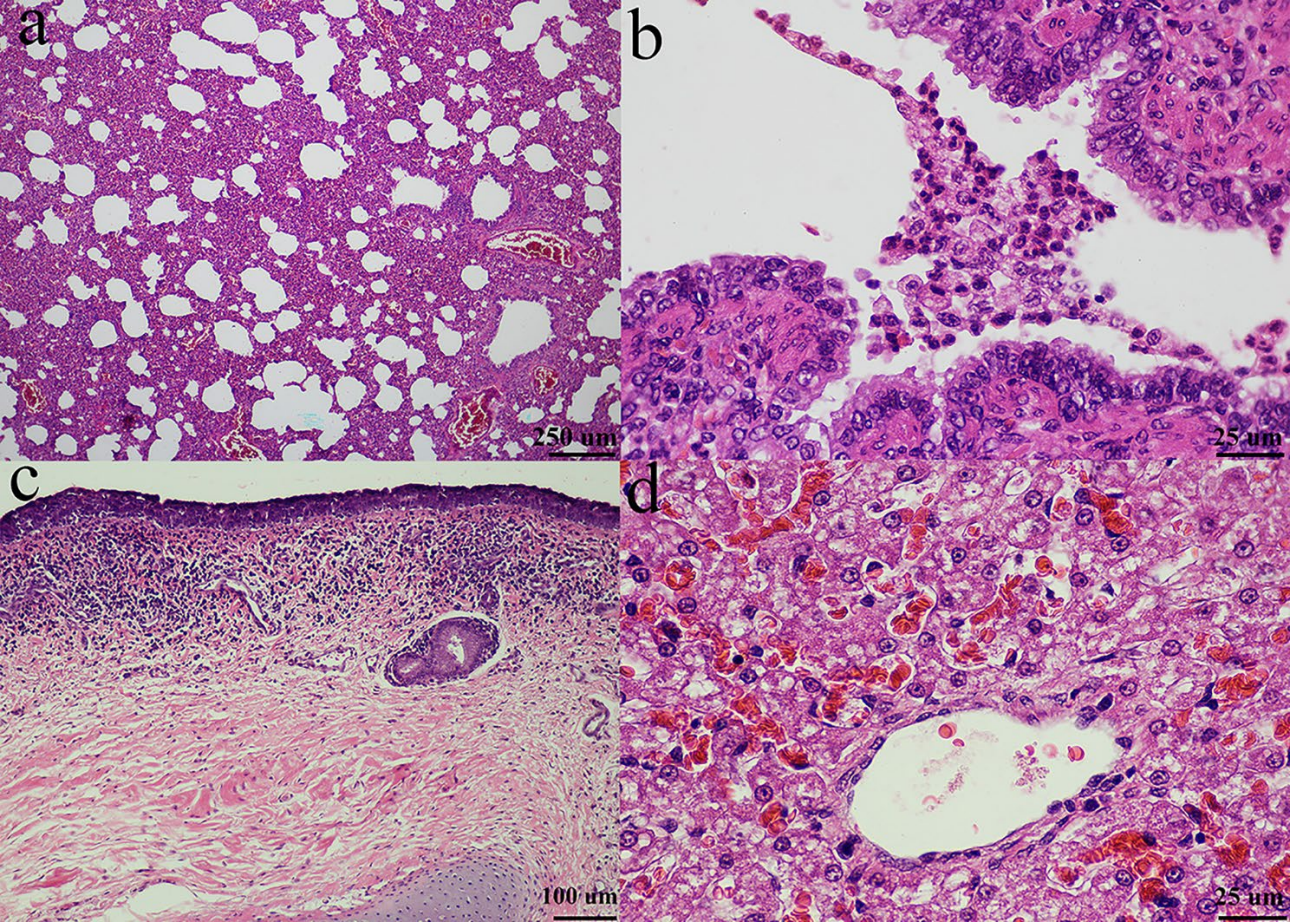
Histopathological changes in CIV-H3N2-challenged dogs, (a) interstitial pneumonia (4x), (b) bronchiolitis obliterans lesion (40x), (c) inflammatory cell infiltration with shortened tracheal epithelium (10x), (d) centrilobular fatty change degeneration (40X)

For pathological changes, dogs in the inoculated group (n = 1) had gross lesions in the lung, spleen and liver at 7 dpi. Lung lobes collapsed with moderate red hepatization. The spleen had a round edge with mild splenomegaly. The liver showed mild hepatic congestion. At 7 dpi in the contact group (n = 1), all lung lobes collapsed with moderate red hepatization and multifocal petechial hemorrhage. At 14 dpi, lung lobes in dogs in both the inoculated group (n = 1) and the contact group (n = 1) showed moderate congestion but no frothy exudate in the tracheal lumen. The histological examination of inoculated dogs and contact dogs at 7 dpi showed diffuse interstitial pneumonia. Pneumocyte type II hyperplasia and inflammatory cells were found. The shortening of tracheal epithelial cells was identified as tracheitis in both groups. At 14 dpi, the inoculated dogs showed moderate diffuse pulmonary edema with focally extensive hemorrhage and mild tracheitis, and the contact dogs showed severe diffuse interstitial bronchopneumonia with edema and moderate tracheitis (Fig. 5).

In conclusion, based on the Thai CIV-H3N2 challenge dog model, the inoculated and direct contact dogs developed respiratory signs at 2 dpi. The dogs shed the virus in the respiratory tract at 1 dpi and developed antibodies against the virus at 10 dpi. Mild lung congestion and histopathological changes in the lung were observed. This result demonstrated dog-to-dog (intraspecies) transmission of Thai CIV-H3N2.

### Transmission and pathogenicity of Thai CIV-H3N2 in guinea pigs

**Fig. 6:**
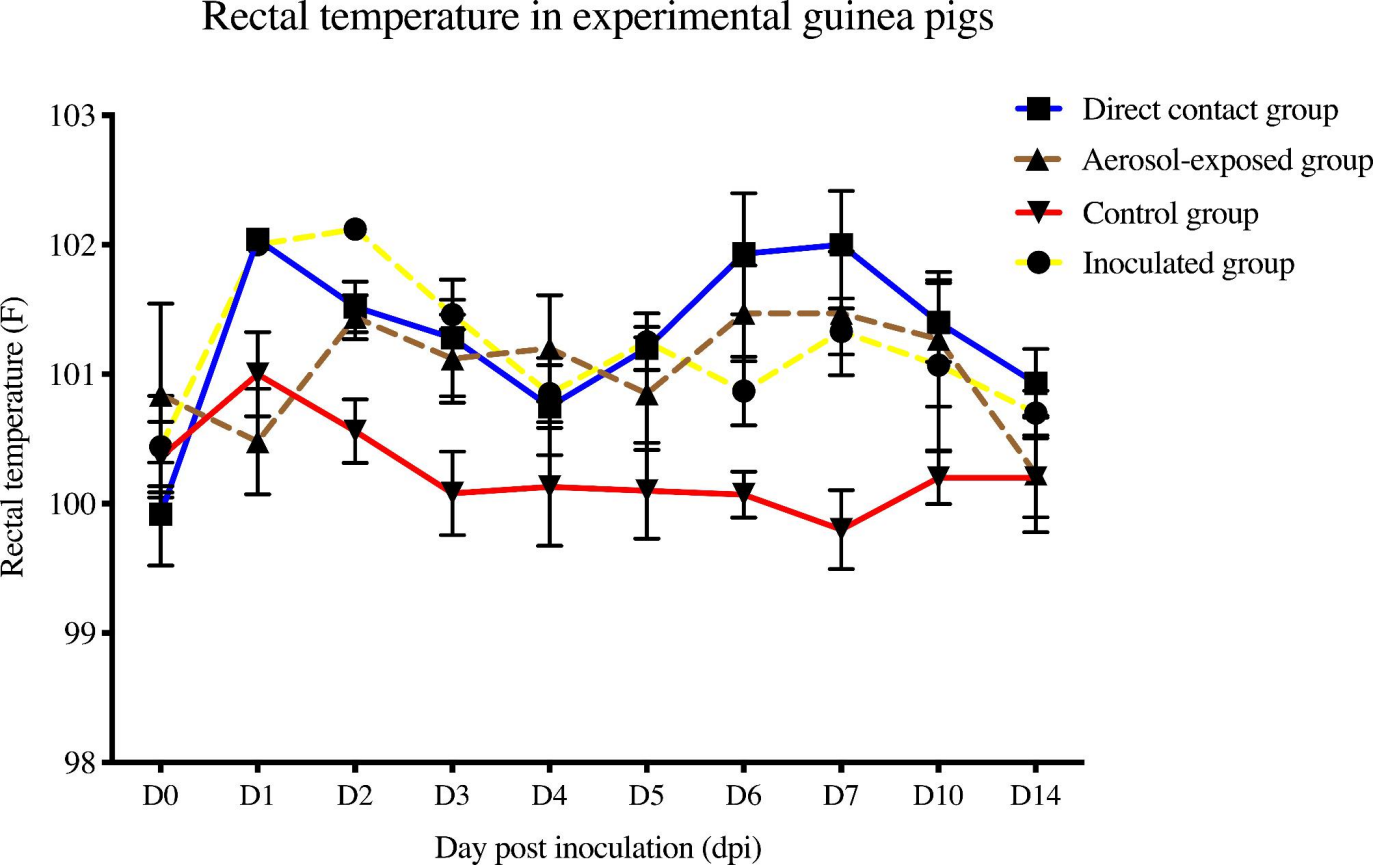
Clinical presentation and body temperature of CIV-H3N2-challenged guinea pigs in the inoculated group, direct contact group, aerosol-exposed group and control group

Guinea pigs of the inoculated group (n = 5) were challenged with 10^6^ EID_50_ of Thai CIV-H3N2. The direct contact group (n = 5) and aerosol-exposed group (n = 5) were placed at 1 dpi. PBS was used as a mock challenge in the control group (n = 5). All guinea pigs in the inoculated group, direct contact group, and aerosol-exposed group showed mild clinical signs and fever compared with the control groups (n = 5) (p < 0.05). The mean weight of the guinea pigs was 497.20-593.67 g in the inoculated group, 468.20-575.33 g in the direct contact group, 490.40–614.67 g in the aerosol-exposed group and 488.00- 612.00 g in the control group, but there was no significant difference among the groups. The body temperature in the inoculated group was 100.44-102.12°F, whereas it was 100.07–101.00°F in the control group. The direct contact and aerosol-exposed groups showed 99.92-102.04°F and 100.23-101.47°F, respectively. The body temperature of guinea pigs in the inoculated group was significantly higher than that in the control group at 1 dpi – 7 dpi (p < 0.05). Similarly, the body temperature of the direct contact and aerosol-exposed groups was significantly higher than that of the control group at 1–10 dpi and at 2–7 dpi, respectively (p < 0.05) (Fig. 6). No significant respiratory signs were observed in guinea pigs, except for hot and red ears due to fever.

**Fig. 7:**
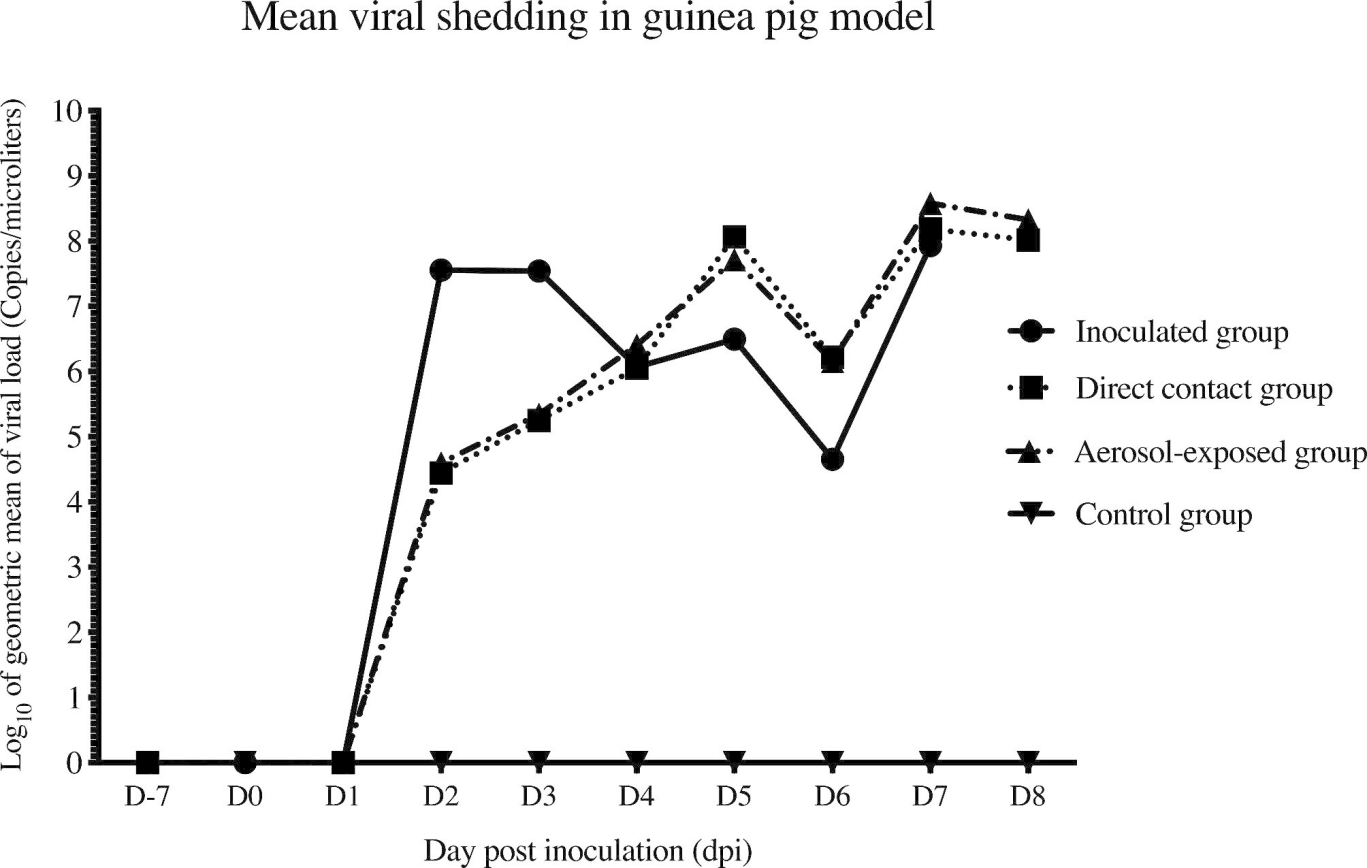
Viral shedding of CIV-H3N2-challenged guinea pigs in the inoculated group, direct contact group, aerosol-exposed group and control group. Viral shedding was presented as log10 of the geometric mean (copies per microliter). Bars represent the standard deviation of the mean viral titer

For viral shedding in guinea pigs, CIV-H3N2 could be detected in the respiratory tract of animals in the inoculated group, direct contact group and aerosol-exposed group. In detail, guinea pigs in the inoculated group shed CIV-H3N2 in the respiratory tract at 2 dpi − 3 dpi. Guinea pigs in the direct contact group and aerosol-exposed group shed the virus from 2 − 3 dpi (equal to 1–2 dpc) with a low viral load (ct > 29) and the viral loads increased at 4–8 dpi (ct < 29). Interestingly, the guinea pigs showed the highest titer in the aerosol-exposed group at 7 dpi. Moreover, the viral titers in the direct contact group and aerosol-exposed group were significantly higher than those in the inoculated group (Fig. 7).

**Fig. 8:**
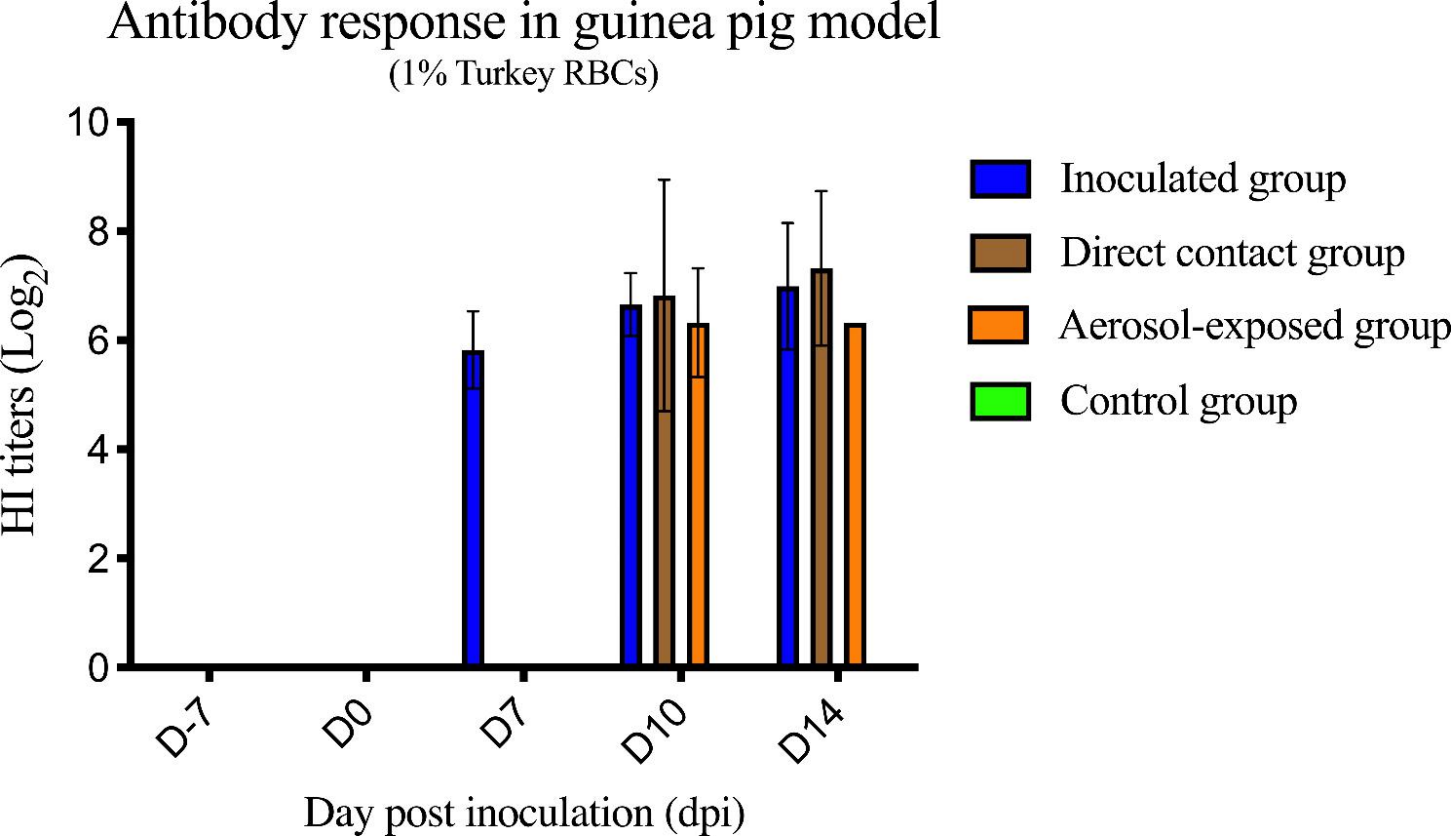
Antibody response (HI test) of CIV-H3N2-challenged guinea pigs in the inoculated group, direct contact group, aerosol-exposed group and control group. The antibody titer was presented as the HI titer. Bars represent the standard deviation of the mean HI titer

For the antibody response to CIV-H3N2, the guinea pigs in the inoculated group (2/3; 66.67%) were seropositive at 7 dpi but guinea pigs in the direct contact (2/3; 66.67%) and aerosol-exposed groups (3/3; 100%) were seropositive at 10 dpi (p < 0.05). These results suggested that HI antibody titers against Thai CIV-H3N2 were completely developed at 10 dpi in the inoculated group (Fig. 8).

**Fig. 9:**
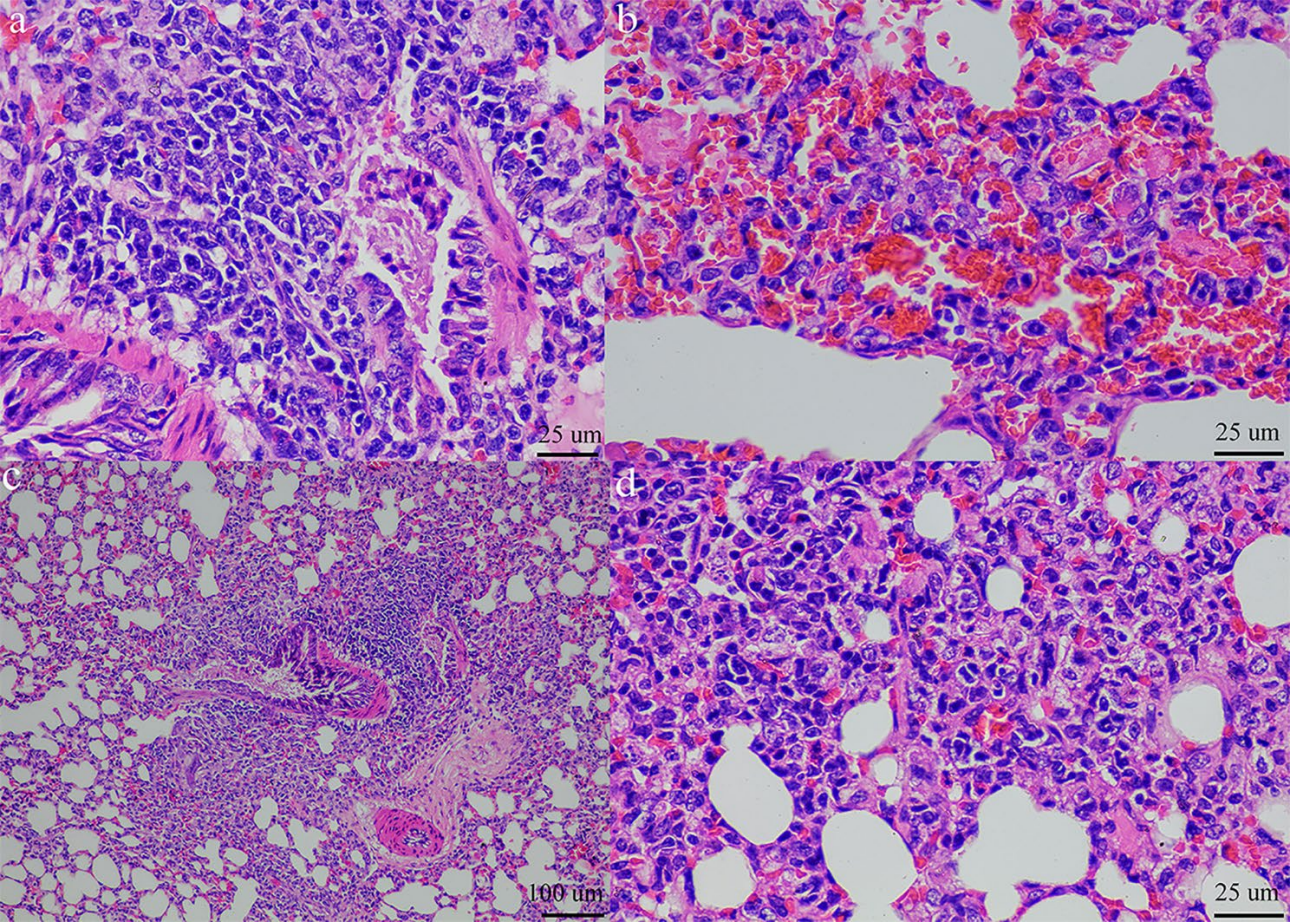
Histopathological changes in CIV-H3N2-challenged guinea pigs, (a) bronchointerstitial pneumonia, BALT hyperplasia and hemorrhage 40x; (b) bronchointerstitial pneumonia, BALT hyperplasia and hemorrhage 40x; (c) bronchointerstitial pneumonia and BALT hyperplasia 10x; (d) interstitial pneumonia and type II pneumocyte hyperplasia 40x

For pathological changes, gross lesions of infected guinea pigs showed mild lung congestion, moderate hepatic congestion and mild splenomegaly. In detail, at 3 dpi, mild congestion in the left caudal lobe of the lung was observed. Moderate emphysema at the periphery was also seen. The spleen was round with mild splenomegaly. The liver had moderate hepatic congestion and the kidney was reddish. Mild lung congestion was also found in the direct contact group and aerosol-exposed group. Histopathological examination in the inoculation group showed necrotizing and lymphocytic bronchointerstitial pneumonia, mild multifocal BALT hyperplasia with type II pneumocyte and PAM hyperplasia with mild tracheitis. In the direct contact and aerosol- exposed groups, the lesions showed bronchointerstitial pneumonia with tracheitis (Fig. 9).

In conclusion, based on the Thai CIV-H3N2 challenge in a guinea pig model, incoculated, direct contact and aerosol-exposed guinea pigs developed fever at 1–2 dpi. The guinea pigs shed the virus in the respiratory tract at 2 dpi and developed antibodies against the virus at 7 dpi. Mild lung congestion and histopathological changes in the lung were observed. In the study, viral shedding and antibody responses were observed in inoculated, direct contact and aerosol-exposed guinea pigs, indicating that Thai CIV-H3N2 isolated from dogs could infect guinea pigs and suggesting possible interspecies transmission of Thai CIV-H3N2.

## Discussion

In this study, the CIV-H3N2 challenge experiment in dogs showed that CIV-H3N2 can infect dogs and cause respiratory signs in both the inoculated and direct contact groups. CIV-H3N2-infected dogs showed clinical signs, including fever, depression, nasal discharge, ocular discharge and coughing. A previous study supported our result that tracheal, bronchial, and bronchiolar epithelial cells of dogs contain both receptors (SAα 2,3-gal and SAα 2,6-gal) for both avian and mammalian viruses, supporting the potential transmission of avian-origin CIV in dogs [12]. In addition, CIV-H3N2-infected dogs can shed the virus from the respiratory tract and develop H3-specific antibodies. In this study, CIV-H3N2 infected and replicated in the respiratory tract of dogs in the inoculated group (100%) beginning at 2 dpi and in the direct contact group beginning at 3 dpi. Interestingly, viral shedding was prolonged until 9 dpi in the inoculated group and 10 dpi in the contact group. Our result was comparable to a previous study in Korea in which dogs infected with CIV-H3N2 (A/canine/Korea/01/2007) began to shed the virus at 1 dpi and continued until 6 dpi [12]. The differences in the viral shedding period among studies can be explained by; (i) the virulence of the virus (Thai CIV-H3N2 and Korea CIV-H3N2) [12, 25] and (ii) the reinfection among dogs in the direct contact group. For the serological results, the infected dogs showed an antibody response against CIV-H3N2 at 10 dpi compared to those in the previous study at 6 and 8 dpi [12].

In this study, we used guinea pigs as an alternate mammalian challenge model [16, 17]. In general, ferrets are used as mammalian models for influenza infection experiments. There have also been many reports of using guinea pigs as models for influenza [17, 19]. Guinea pigs are suitable for both large droplet and airborne viral transmission in mammalian hosts [18]. They are susceptible to both avian and human influenza viruses and are very useful for influenza virus transmission studies. However, the immunological response to influenza in guinea pigs is unclear because of the paucity of species-specific reagents [26].

The CIV-H3N2 challenge study in a guinea pig model showed that CIV-H3N2 can infect guinea pigs and transmit to other guinea pigs through both direct contact and aerosol exposure, confirming guinea pig-to-guinea pig transmission. The guinea pigs in the inoculated group shed the virus beginning at 2 dpi. The nasal sheding in the inoculated group was highest at 2–3 dpi, similar to a previous study in which viral sheding in the nasal cavity was highest at 3 dpi after infecting guinea pigs with H1 and H3 swine influenza virus [17]. Our result is also in agreement with a previous study showing that guinea pigs challenged with pandemic H1N1-2009 shed the virus beginning at 2 dpi [27]. Intermittent viral shedding was observed in guinea pigs in all groups. For the serological results, the guinea pigs possessed H3 antibodies against Thai CIV-H3N2 beginning at 7 dpi and 100% at 10 dpi in the inoculated group. In the contact group, guinea pigs presented an antibody response starting at 10 dpi. Our results demonstrated that CIV-H3N2 can induce an antibody response in guinea pigs. It has been reported that SAα 2,3-gal and SAα 2,6-gal receptors are widely present in the nose and trachea of guinea pigs and the SAα 2,3-gal receptor is dominantly present in the lung; thus, infection and an antibody response to CIV-H3N2 in guinea pigs is possible [28].

Our result raises the possibility of the transmission of canine influenza from dogs or intermediate mammals (guinea pigs) to humans, since both SAα 2,3-gal and SAα 2,6-gal receptors are also present in the human respiratory tract. A previous study on sialic acid receptor binding showed that the mutation of HA (Q226L, Q226R, and G228S) increases the binding preference and binding affinity of H3N2 influenza viruses for human-type receptors ([29]. Thai CIV-H3N2 dose not contain HA mutations at Q226 or G228, indicating a possible lower binding affinity of the virus for the mammalian receptor. Unfortunately, we did not perform sialic acid receptor binding analysis on Thai CIV-H3N2 to confirm its binding affinity. To date, there are no reports of CIV-H3N2 infection in humans; however, the reassortment of the viruses to novel or more virulent forms should not be ignored. The reassortment of CIV-H3N2 was reported in a previous study [30]. The reassortment of contemporary influenza viruses can result in higher viral replication transmissibility and virulence [31, 32]. Moreover, dog breed can also contribute to a more human-animal interface and lead to viral spillover [33].

## Conclusion

In this study, the intraspecies transmission of Thai CIV-H3N2 was demonstrated in experimentally challenged dogs. The infected dogs in the inoculated and direct contact groups presented clinical signs, such as fever, serous nasal discharge, ocular discharge, coughing, depression and loss of appetite. All dogs could shed the virus and had an antibody response against CIV-H3N2. The interspecies transmission of CIV-H3N2 was demonstrated in experimentally challenged guinea pigs. The infected guinea pigs showed fever and mild clinical signs related to respiratory disease. Guinea pigs can shed the virus and develop H3-specific antibodies. In Thailand, there are no recommendations for canine influenza vaccines used in pet animals. The canine influenza vaccine for dogs has been developed and used in Korea and China. The inactivated A/canine/Korea/01/07 (H3N2) was shown to be highly efficient in reducing fever and lung lesions and decreasing viral shedding in dogs [34]. In addition, the live-attenuated vaccine was developed and showed higher immunogenicity and protective efficacy than the inactivated influenza vaccine [35]. Canine influenza vaccination in dogs will be another option for the prevention and control of canine influenza virus among dogs and minimize transmission. Moreover, the personal hygiene of pet owners and animal health care workers should be regularly practiced to avoid close contact transmission. One health approach can help raise awareness of the human-domestic animal interface contributing to the potential zoonotic transmission of influenza.

## Data Availability

The nucleotide sequence data that support the findings of this study are openly available in GenBank at http://www.ncbi.nlm.nih.gov/genbank/, A/canine/Thailand/CU-DC5299/2012 (H3N2) (KC599545-52).
